# Predictors of cervical myelopathy and hydrocephalus in young children with achondroplasia

**DOI:** 10.1186/s13023-021-01725-4

**Published:** 2021-02-12

**Authors:** Youngbo Shim, Jung Min Ko, Tae-Joon Cho, Seung‐Ki Kim, Ji Hoon Phi

**Affiliations:** 1 Division of Pediatric Neurosurgery, Seoul National University Children’s Hospital, Seoul National University College of Medicine, 101 Daehak-ro, Jongno-gu, 03080 Seoul, Republic of Korea; 2 Department of Pediatrics, Seoul National University Children’s Hospital, Seoul National University College of Medicine, Seoul, Republic of Korea; 3 Division of Pediatric Orthopaedics, Seoul National University Children’s Hospital, Seoul, Republic of Korea; 4 Department of Orthopaedic Surgery, Seoul National University College of Medicine, Seoul, Republic of Korea

**Keywords:** Achondroplasia, Cervical myelopathy, Foramen magnum decompression, Hydrocephalus, Predictor, Ventriculo-peritoneal shunt

## Abstract

**Background:**

Cervical myelopathy and hydrocephalus occasionally occur in young children with achondroplasia. However, these conditions are not evaluated in a timely manner in many cases. The current study presents significant predictors for cervical myelopathy and hydrocephalus in young children with achondroplasia.

**Methods:**

A retrospective analysis of 65 patients with achondroplasia who visited Seoul National University Children’s Hospital since 2012 was performed. The patients were divided into groups according to the presence of cervical myelopathy and hydrocephalus, and differences in foramen magnum parameters and ventricular parameters by magnetic resonance imaging between groups were analyzed. Predictors for cervical myelopathy and hydrocephalus were analyzed, and the cut-off points for significant ones were calculated.

**Results:**

The group with cervical myelopathy showed foramen magnum parameters that indicated significantly lower cord thickness than in the group without cervical myelopathy, and the group with hydrocephalus showed significantly higher ventricular parameters and ‘Posterior indentation’ grade than the group without hydrocephalus. ‘Cord constriction ratio’ (OR 5199.90, p = 0.001) for cervical myelopathy and ‘Frontal horn width’ (OR 1.14, p = 0.001) and ‘Posterior indentation’ grade (grade 1: OR 9.25, p = 0.06; grade 2: OR 18.50, p = 0.01) for hydrocephalus were significant predictors. The cut-off points for cervical myelopathy were ‘Cord constriction ratio’ of 0.25 and ‘FM AP’ of 8 mm (AUC 0.821 and 0.862, respectively) and ‘Frontal horn width’ of 50 mm and ‘Posterior indentation’ grade of 0 (AUC 0.788 and 0.758, respectively) for hydrocephalus.

**Conclusion:**

‘Cord constriction ratio’ for cervical myelopathy and ‘Frontal horn width’ and ‘Posterior indentation’ grade for hydrocephalus were significant predictors and may be used as useful parameters for management. ‘Posterior indentation’ grade may also be used to determine the treatment method for hydrocephalus.

## Background

Achondroplasia is the most common form of dwarfism, occurring in 1 in 26,000 live-born infants [1]. High cervical myelopathy occasionally occurs in young children with achondroplasia [2, 3]. Although the risk of unexpected death due to craniocervical constriction decreases after the first year [1, 4, 5], the small size of the foramen magnum does not change, which may increase the likelihood of insidious or accidental cord injury. T2 high signal intensity (HSI) in the cervical cord is observed on magnetic resonance imaging (MRI) in 40% of asymptomatic achondroplasia patients [6–8].

Approximately 4.3% of achondroplasia patients require ventriculo-peritoneal (VP) shunts [9]. Ventriculomegaly may be observed in asymptomatic patients [10]. Symptoms of acute hydrocephalus rarely occur, but tense fontanel, prominence of superficial venous patterning, lethargy, and irritability do appear insidiously. Unlike other hydrocephalus patients, the size of the cranial base, which is formed by endochondral ossification, is smaller, and the jugular foramen is also smaller. This condition causes a partial obstruction of venous flow, which increases intracranial venous pressure and causes problems with the venous resorption of cerebrospinal fluid (CSF) [11–13]. CSF flow obliteration at the craniocervical junction has also been suggested as a factor in some studies [14], and a VP shunt is considered the standard treatment.

Cervical myelopathy and hydrocephalus may occur in young children with achondroplasia. However, these conditions are not evaluated in a timely manner, and the outcomes lead to permanent neurological deficits in patients. Therefore, we identified significant predictors for cervical myelopathy and hydrocephalus in young children with achondroplasia.

## Materials and methods

### Patients

**Fig. 1:**
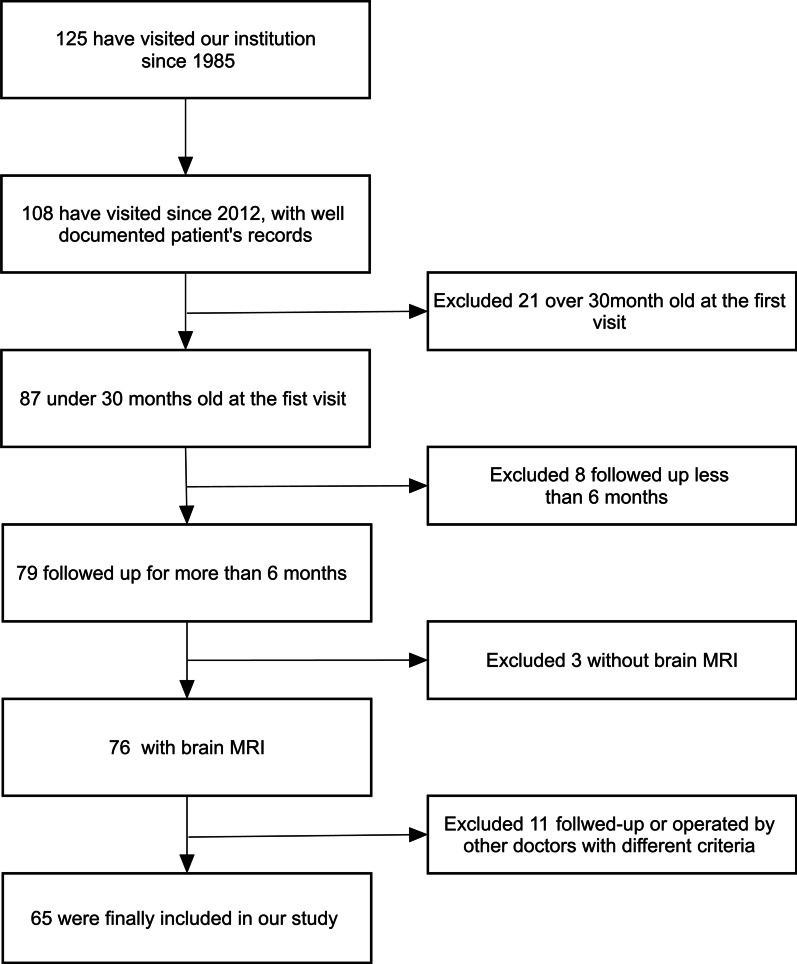
Flowchart of the patients

Since 1985, 125 patients with achondroplasia visited Seoul National University Children’s Hospital. A total of 108 patients visited since 2012, with well-documented patient records. We excluded older patients (> 30 months) because most of them were outside the vulnerable period for cervical myelopathy or had already received surgical procedures at other institutions. Eighty-seven patients were under 30-months-old at the first visit. We included 79 patients who were followed for more than 6 months. Three patients for whom MRI was not performed were excluded. To apply consistent evaluation criteria, 65 patients who were continuously managed by a single pediatrician and a single neurosurgeon were ultimately included (Fig. 1). All 65 patients were clinically diagnosed with a de novo case of achondroplasia without family history and confirmed to have the *FGFR3* NP_000133.1:p.(Gly380Arg) mutation. The identification of a heterozygous pathogenic variant in *FGFR3* using molecular genetic testing establishes the diagnostic confirmation of achondroplasia. Because a common pathogenic variant, p.Gly380Arg, is identified in approximately 98% of patients with achondroplasia, we first performed targeted Sanger analysis [15, 16] of the ‘mutation-hot spot’ p.Gly380Arg and detected this common mutation in all patients who participated in the study. DNA was extracted from peripheral blood leukocytes from each patient and amplified using PCR and a primer pair of *FGFR3* exon 8. Direct Sanger sequencing was subsequently performed using amplified PCR products and revealed a common pathogenic mutation, p.Gly380Arg, in all participants.

### Criteria for the diagnosis and treatment of cervical myelopathy and hydrocephalus

MRI for spinal cord injury was considered for all patients at 6 months of age, but this screening was infrequently used because many of the patients were well over 6 months of age when they visited our institution. The treatment plan was established based on the symptoms of the patients and the MRI findings at the first visit and follow-up.

Cervical myelopathy was diagnosed when there were (1) neurological abnormalities, including long tract sign, apnea, or a history of respiratory arrest or (2) evidence of spinal cord injury (HSI on T2 images of MRI). We performed foramen magnum decompression (FMD) when patients had (1), (2), or (3) severe indentation (50% or greater than normal cord thickness) of the cervical spinal cord on the initial MRI. Except for the two initial patients, C1 laminectomy was also performed with FMD. Intraoperative neurophysiological monitoring was performed in all patients to prevent spinal cord injury, and decompression was performed sufficiently larger than the cord width as measured on preoperative MRI.

Hydrocephalus was diagnosed when there were (1) overt neurological abnormalities, such as irritability, eyeball movement limitation, or sunset sign, (2) abnormalities on physical examination, such as rapid increases in head circumference (HC), tense fontanel, or scalp vein engorgement, accompanied by (3) overt ventriculomegaly with periventricular T2 HSI on MRI. A VP shunt was indicated when symptoms and signs were severe, such as rapidly increasing ventricular size; FMD was performed when the symptoms and signs were not severe, and the risk of cervical myelopathy was likely to be high, considering the long-term complications of VP shunts. These decisions were based on clinical assessments but were not determined using objective parameters.

### Parameters for predicting cervical myelopathy and hydrocephalus

**Fig. 2:**
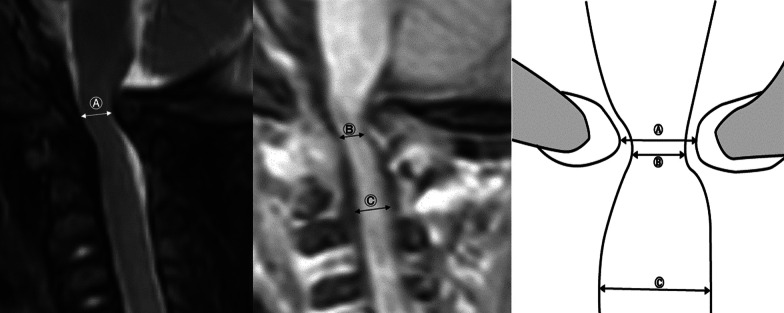
‘FM AP’ (A) was measured using T2 sagittal MRI images. The T1 sagittal MRI image was used to measure ‘Cord thickness narrowest’ (B) and ‘Cord thickness C2’ (C). We created a new parameter called ‘Cord constriction ratio’ and calculated it as ‘1—(B/C)’

Using the T2 sagittal image of MRI, the anterior–posterior diameter of the foramen magnum (FM AP), excluding the soft tissue portion, of the symptomatic group and the asymptomatic group was measured, and the cord signal change was checked (Fig. 2). Using the T1 sagittal image of MRI, the cord thickness at the level of the foramen magnum (Cord thickness FM), the narrowest cord thickness between the foramen magnum and C2 (Cord thickness narrowest), and the cord thickness at the level of C2 (Cord thickness C2) were measured.

**Fig. 3:**
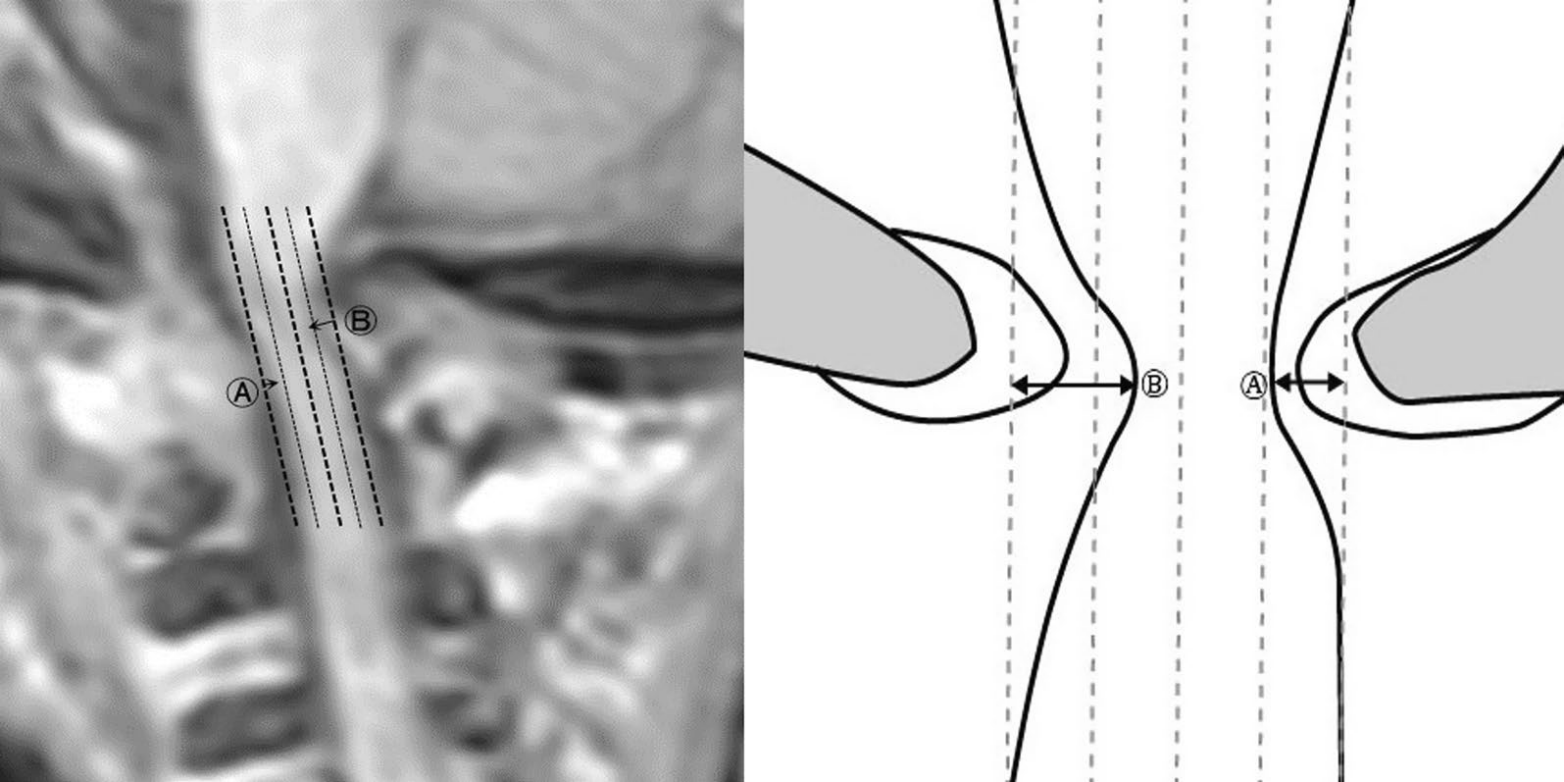
After the cord was divided longitudinally into 4 sections based on ‘Cord thickness C2’, cord indentation was graded (grade 1: < 25%, A; grade 2: ≥ 25%, B) at the anterior and posterior parts. The ‘Indentation index’ was calculated by adding the grades of the ‘Anterior indentation’ and ‘Posterior indentation’

We created four new parameters: ‘Cord constriction ratio’, ‘Anterior indentation’, ‘Posterior indentation’, and ‘Indentation index’. The ‘Cord constriction ratio’ was the value obtained by ‘1—(‘Cord thickness narrowest’/‘Cord thickness C2’ which is the normal part)’. Cord indentation was graded from the anterior and posterior sides after the cord was divided into 4 parts in the longitudinal direction based on ‘Cord thickness C2’ (grade 0: no indentation; grade 1: below 25%; grade 2: 25% or above) (Fig. 3). The ‘Indentation index’ was obtained by adding the grades of the ‘Anterior indentation’ and ‘Posterior indentation’. ‘Frontal horn width’, ‘Evans ratio’, and ‘Periventricular HSI’ (mm^2^) were also measured using T2 axial images.

### Statistical analysis

We retrospectively compared differences in the foramen magnum (FM) parameters (‘FM AP’, ‘Cord constriction ratio’, ‘Anterior indentation’, ‘Posterior indentation’, and ‘Indentation index’) and ventricular parameters (‘Frontal horn width’, ‘Evans ratio’, and ‘Periventricular HSI’) according to the presence of cervical myelopathy and hydrocephalus using independent T-tests and Fisher’s exact test.

Logistic regression analyses for cervical myelopathy and hydrocephalus were performed using the parameters described above to identify significant predictors. Univariate analyses using each parameter were performed for cervical myelopathy and hydrocephalus. Because many parameters were highly interrelated (e.g., when ‘FM AP’ was lower, ‘Cord constriction ratio’ was higher in most instances), we selected relatively more significant parameters from FM parameters and ventricular parameters. Basic parameters (age, sex) were added to create a generalized linear model. Multivariate analysis was performed with the constructed model without multicollinearity.

A receiver operating characteristic (ROC) curve was drawn using the significant predictors to calculate the most appropriate cut-off point. Significant findings were based on a P value of 0.05 or less. All statistical analyses were performed using R version 4.0.0. The Institutional Review Boards of our institution approved this study, and patient consent was not required.

## Results

### Patients’ characteristics

Thirteen (20%) of the 65 patients showed T2 HSI on MRI, long tract sign, or respiratory difficulty that corresponded to cervical myelopathy, and 8 patients (12%) had symptoms or signs related to hydrocephalus, such as scalp vein engorgement, tense fontanel, and papilledema. Four (6%) patients had symptoms of cervical myelopathy and hydrocephalus. Twenty-one patients (32%) underwent FMD, and 1 patient (2%) had a VP shunt. Six of the patients who underwent FMD (9%) had no symptoms or signs of cervical myelopathy or hydrocephalus but did have severe cord indentation, a low ‘FM AP’, and a high ‘Cord constriction ratio’, which led to preventive surgery. The mean follow-up period for all patients was 44 months, and it was 33 months for patients who underwent surgery.

### Differences in FM parameters and ventricular parameters according to patient status

Comparison of the patient characteristics and measurements according to the presence of cervical myelopathy or hydrocephalus (Tables 1 and 2) revealed that the patients with cervical myelopathy showed a significantly lower ‘FM AP’ (p < 0.001) and higher ‘Cord constriction ratio’, ‘Anterior indentation’, ‘Posterior indentation’ grade, and ‘Indentation index’ than patients without cervical myelopathy (all p < 0.001). Notably, there was no cervical myelopathy in the absence of indentation.

**Table 1:** Differences in patient characteristics and measurements according to the presence of cervical myelopathy

Characteristics and parameters^a^	Yes	No	p value
N	13	52	
Sex			
Male	8 (62)	28 (54)	0.85
Female	5 (38)	24 (46)	
Age (months)	11.7 ± 6.1	14.6 ± 9.3	0.29
Follow up period (months)	31.0 ± 22.3	41.8 ± 23.8	0.15
FM AP (mm)	6.06 ± 1.66	9.10 ± 2.22	< 0.001
Cord constriction ratio	0.37 ± 0.19	0.16 ± 0.12	< 0.001
Anterior indentation			
0	0 (0)	24 (46)	< 0.001
1	3 (23)	21 (40)	
2	10 (77)	7 (14)	
Posterior indentation			
0	0 (0)	38 (73)	< 0.001
1	7 (54)	8 (15)	
2	6 (46)	6 (12)	
Indentation index			
0	0 (0)	22 (42)	< 0.001
1	0 (0)	15 (29)	
2	3 (23)	8 (15)	
3	4 (31)	4 (8)	
4	6 (46)	3 (6)	
Frontal horn width (mm)	43.94 ± 11.04	40.61 ± 7.88	0.22
BPD (mm)	141.17 ± 7.46	136.40 ± 8.34	0.06
Evans ratio	0.31 ± 0.07	0.30 ± 0.06	0.51
Periventricular HSI	22.09 ± 20.33	11.46 ± 11.92	0.02
FMD			
Yes	12 (92)	9 (17)	
No	1 (8)	43 (83)	
VP shunt			
Yes	0 (0)	1 (2)	
No	13 (100)	51 (98)	
Later event			
Yes	0 (0)	2 (4)	1.00
No	13 (100)	50 (96)	

*FM* Foramen magnum, *AP* anterior–posterior diameter, *BPD* bi-parietal diameter, *HSI* high signal intensity, *FMD* foramen magnum decompression, *VP* ventriculo-peritoneal

^a^Data are presented as n (%) or mean ± SD

**Table 2:** Differences in patient characteristics and measurements according to the presence of hydrocephalus

Characteristics and parameters^a^	Yes	No	p-value
N	8	57	
Sex			
Male	5 (63)	31 (54)	0.96
Female	3 (37)	26 (46)	
Age (months)	7.25 ± 4.20	14.98 ± 8.92	0.02
Follow up period (months)	18.12 ± 17.90	42.63 ± 22.99	0.005
FM AP (mm)	6.78 ± 1.90	8.73 ± 2.42	0.03
Cord constriction ratio	0.28 ± 0.14	0.19 ± 0.16	0.145
Anterior indentation			
0	3 (38)	21 (37)	0.20
1	1 (12)	23 (40)	
2	4 (50)	13 (23)	
Posterior indentation			
0	1 (12)	37 (65)	0.009
1	3 (38)	12 (21)	
2	4 (50)	8 (14)	
Indentation index			
0	1 (12)	21 (37)	0.19
1	1 (12)	14 (25)	
2	2 (25)	9 (16)	
3	1 (12)	7 (12)	
4	3 (38)	6 (10)	
Frontal horn width (mm)	49.85 ± 11.70	40.08 ± 7.46	0.002
BPD (mm)	138.96 ± 13.40	137.13 ± 7.53	0.57
Evans ratio	0.36 ± 0.08	0.29 ± 0.05	0.002
Periventricular HSI (mm^2^)	22.49 ± 22.98	12.34 ± 12.65	0.06
FMD			
Yes	7 (88)	14 (25)	
No	1 (12)	43 (75)	
VP shunt			
Yes	1 (12)	0 (0)	
No	7 (88)	57 (100)	
Later event			
Yes	0 (0)	2 (3)	1.00
No	8 (100)	55 (97)	

*FM* Foramen magnum, *AP* anterior–posterior diameter, *BPD* bi-parietal diameter, *HSI* high signal intensity, *FMD* foramen magnum decompression, *VP* ventriculo-peritoneal

^a^Data are presented as n (%) or mean ± SD

Nine patients had no cervical myelopathy, but FMD was performed. They had grade 1 or 2 ‘Posterior indentation’ with a narrow cord thickness (‘Cord thickness narrowest’: 3.31–5.33 mm). In contrast, 1 patient did not undergo FMD despite cord injury. T2 HSI was observed in the cervical spinal cord on MRI, but the patient had no symptoms, and the FM parameters were not bad overall (‘FM AP’: 9.10 mm; ‘Cord thickness narrowest’: 5.74 mm; ‘Cord constriction ratio’: 0.07; ‘Anterior indentation’: grade 1; and ‘Posterior indentation’: grade 1). The patient was already 25 months old at the time of the outpatient visit and was thought to have had a spinal cord injury when she was younger. The patient had been doing well without any problems as the foramen magnum widened.

The patients with hydrocephalus had a significantly higher ‘Frontal horn width’ and ‘Evans ratio’ (both p = 0.002) than patients without hydrocephalus. None of the FM parameters, except ‘Posterior indentation’, showed significant differences, but ‘Posterior indentation’ was significantly different (p = 0.009). In the absence of ‘Posterior indentation’, the probability of having hydrocephalus was only 3% (1 of 38). However, the probability with grade 1 ‘Posterior indentation’ was 20% (3 of 15), and the probability with grade 2 was 33% (4 of 12).

Except for two patients, all (97%) who underwent surgery or observed according to our criteria did well without any events. Later events, such as any neurological symptoms or signs that occurred during follow-up, only occurred in 2 patients who were followed without surgery. One of these two patients tended to have better values than the other patients with cervical myelopathy (‘FM AP’: 8.52 mm; ‘Cord thickness narrowest’: 5.75 mm; ‘Cord constriction ratio’: 0.03; ‘Anterior indentation’: grade 1; and ‘Posterior indentation’: grade 0), but temporary respiratory difficulty did occur after the patient slipped and fell during follow-up. At the time of the outpatient visit, she had already recovered and did not need further treatment. The other patient also had better values than the other patients with cervical myelopathy (‘FM AP’: 10.01 mm; ‘Cord thickness narrowest’: 5.08 mm; ‘Cord constriction ratio’: 0.19; ‘Anterior indentation’: grade 0; and ‘Posterior indentation’: grade 0), but ventriculomegaly was present without obvious symptoms of hydrocephalus. During follow-up, he presented delayed speech development and an increased ventricular size, which led to surgery.

### Identification of predictors for cervical myelopathy and hydrocephalus

#### Predictors for cervical myelopathy

Based on the univariate analyses for the identification of predictors for cervical myelopathy, ‘FM AP’ and ‘Cord constriction ratio’ were significant (all p = 0.001), and the odds ratios (ORs) of these parameters tended to increase as ‘FM AP’ decreased or ‘Cord constriction ratio’ increased (Table 3). Basic parameters (sex, age), ‘Cord constriction ratio’, which was the most significant of the FM parameters, and ‘Frontal horn width’, which was the most significant of the ventricular parameters in univariate analyses, were used to create the generalized linear model for cervical myelopathy. Indentation parameters (‘Anterior indentation’, ‘Posterior indentation’, and ‘Indentation index’) were not included in the model because these factors were not significant in univariate analysis and showed multicollinearity with ‘Cord constriction ratio’. Multivariate logistic regression using the model revealed ‘Cord constriction ratio’ as an important predictor for cervical myelopathy, which was similar to the univariate analysis (OR 4059.18, p = 0.002).

**Table 3:** Univariate and multivariate logistic regression analysis of parameters for cervical myelopathy

Parameter	Univariate analysis			Multivariate analysis		
	OR	95% CI	p value	OR	95% CI	p value
Sex: female	0.73	0.21–2.53	0.62	1.01	0.21–4.79	0.99
Age	0.95	0.86–1.05	0.29	0.98	0.88–1.10	0.77
FM AP	0.46	0.29–0.73	0.001			
Cord constriction ratio	5199.90	31.64–854,573.57	0.001	4059.18	23.04–715,067.58	0.002
Anterior indentation grade 1		0.00-Inf	0.99			
Anterior indentation grade 2		0.00-Inf	0.99			
Posterior indentation grade 1		0.00-Inf	0.99			
Posterior indentation grade 2		0.00-Inf	0.99			
Indentation index 1		0.00-Inf	1.00			
Indentation index 2		0.00-Inf	1.00			
Indentation index 3		0.00-Inf	1.00			
Indentation index 4		0.00-Inf	1.00			
Frontal horn width	1.04	0.97–1.12	0.22	1.01	0.93–1.11	0.77
Evans ratio	32.25	0.00–808,255.26	0.50			

*OR* Odds ratio, *CI* confidence interval, *FM* foramen magnum, *AP* anterior–posterior diameter, *Inf* Infinite

#### Predictors for hydrocephalus

Based on univariate analyses for the identification of predictors for hydrocephalus, ‘Frontal horn width’ and ‘Evans ratio’ were significant (p = 0.001 and 0.01, respectively), as expected, and ‘Posterior indentation’ among the FM parameters was surprisingly statistically significant (grade 1: OR 9.25, p = 0.06; grade 2: OR 18.50, p = 0.01) (Table 4). Multivariate logistic regression analysis was performed with basic parameters (sex, age), ‘Frontal horn width’, and ‘Posterior indentation’, and ‘Frontal horn width’ showed statistical significance (OR 1.16, p = 0.05), and ‘Posterior indentation’ also showed a certain trend toward significance (grade 1: OR 19.72, p = 0.08; grade 2: OR 14.88, p = 0.08), which was similar to univariate analyses.

**Table 4:** Univariate and multivariate logistic regression analysis of parameters for hydrocephalus

Parameter	Univariate analysis			Multivariate analysis		
	OR	95% CI	p value	OR	95% CI	p value
Sex: female	0.72	0.16–3.28	0.67	4.65	0.27–78.89	0.29
Age	0.67	0.50–0.90	0.008	0.74	0.54–1.00	0.05
FM AP	0.69	0.48–0.99	0.05			
Cord constriction ratio	0.05	0.00–3.15	0.15			
Anterior indentation grade 1	0.30	0.03–3.16	0.32			
Anterior indentation grade 2	2.15	0.41–11.20	0.36			
Posterior indentation grade 1	9.25	0.88–97.47	0.06	19.72	0.72–543.40	0.08
Posterior indentation grade 2	18.50	1.82–188.39	0.01	14.88	0.73–303.86	0.08
Indentation index 1	1.50	0.09–36.01	0.78			
Indentation index 2	4.67	0.37–58.24	0.23			
Indentation index 3	3.00	0.16–54.56	0.45			
Indentation index 4	10.05	0.92–120.24	0.06			
Frontal horn width	1.14	1.03–1.26	0.001	1.16	1.00–1.35	0.05
Evans ratio	159,603,019	63.5-Inf	0.01			

*OR* Odds ratio, *CI* confidence interval, *FM* foramen magnum, *AP* anterior–posterior diameter, *Inf* infinite

### Identification of the most appropriate cut-off points of predictors using the ROC curves

#### Cervical myelopathy (Table 5, Fig. 4)

**Table 5:** The cut-off points calculated using ROC curves and its sensitivity, specificity and AUC

Predictor	Cut-off point	Sensitivity (%)	Specificity (%)	AUC
Cervical myelopathy				
FM AP	8.02 mm	92.3	69.2	0.862
Cord constriction ratio	0.25	76.9	80.8	0.821
Hydrocephalus				
Posterior indentation	Grade 0	87.5	64.9	0.788
Frontal horn width	50.63 mm	75.0	91.2	0.758

*ROC* Receiver operating characteristics, *AUC* area under the ROC curve, *FM* foramen magnum, *AP* anterior–posterior diameter

**Fig. 4:**
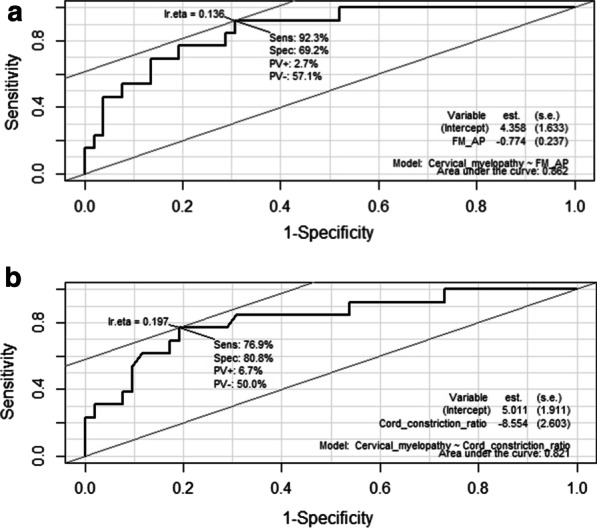
ROC curve for predicting cervical myelopathy using ‘FM AP’ (**a**) and ‘Cord constriction ratio’ (**b**)

The most appropriate cut-off points for ‘FM AP’ and ‘Cord constriction ratio’ were 8.02 mm and 0.25, respectively. ‘FM AP’ and ‘Cord constriction ratio’ showed high AUCs (0.862 and 0.821, respectively). ‘FM AP’ was relatively sensitive (92.3%), and ‘Cord constriction ratio’ was relatively specific (80.8%). Notably, none of the patients with an ‘FM AP’ of 8 mm or greater needed FMD. When the specificity was greater than 95%, the highest ‘FM AP’ was 5.5 mm (Specificity 96%, Sensitivity 46%) and the lowest ‘Cord constriction ratio’ was 0.40 (Specificity 96%, Sensitivity 31%).

#### Hydrocephalus (Table 5, Fig. 5)

**Fig. 5:**
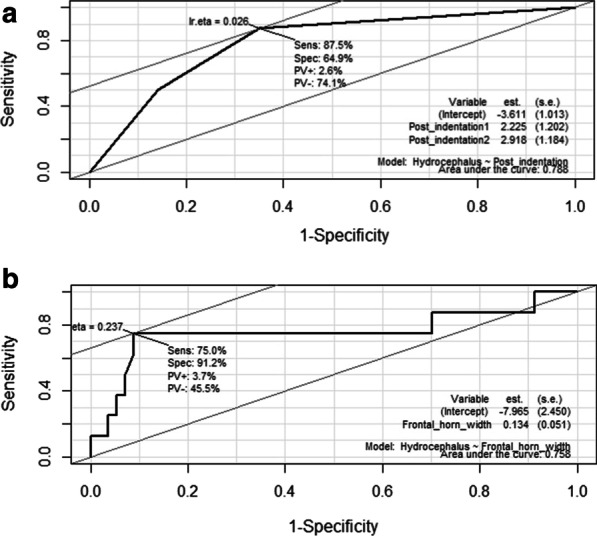
ROC curve for predicting hydrocephalus using ‘Posterior indentation’ (**a**) and ‘Frontal horn width’ (**b**)

The most appropriate cut-off points for ‘Posterior indentation’ and ‘Frontal horn width’ were grade 0 and 50.63 mm, respectively. ‘Posterior indentation’ and ‘Frontal horn width’ showed high AUCs (0.788 and 0.758, respectively). ‘Posterior indentation’ was relatively sensitive (87.5%), and ‘Frontal horn width’ was relatively specific (91.2%). ‘Frontal horn width’ showed the expected results, but it was surprising that ‘Posterior indentation’ was a good predictor for hydrocephalus.

### The practical guideline for FMD based on our study

Based on the above analyses, we established a practical guideline for FMD: (1) symptoms or signs of cervical myelopathy; (2) evidence of cervical spinal cord injury (T2 HSI on MRI); (3) significant cervical spinal cord compression (absolute indication: ‘FM AP’ ≤ 5.5 mm or ‘Cord constriction ratio’ ≥ 0.40; relative indication: ‘FM AP’ ≤ 8.0 mm or ‘Cord constriction ratio’ ≥ 0.25); and (4) ‘Posterior indentation’ in chronic hydrocephalus with mild symptoms, in which VP shunt should be considered when symptoms are acute and severe.

## Discussion

Twenty-two of the 65 patients (34%) in our study underwent surgery, which is a rate somewhat higher than in other papers (9.4–11.7%) [5, 9]. This difference may be the result of the referral of relatively severe cases to our institution. Considering the rates of later events in the patients who underwent surgery (0%, 0 of 22) and who were observed (5%, 2 of 43), the surgical indication was properly applied to the patients, but it may suggest that the surgical indications should be broadened.

### The use of parameters as predictors for cervical myelopathy

Our achondroplasia patients underwent their first MRI at 14.0 months on average. If only those with cervical myelopathy were analyzed, the patients underwent their first MRI at 11.7 months on average. If cord injury had already occurred, neurological deficits remained even after FMD. Therefore, we needed an appropriate MRI screening for achondroplasia patients and significant predictors that may be used to observe progression or determine preventive surgery. However, there was no clear consensus on this issue [17].

We suggest the following guidelines based on the results of our research. First, we recommend performing the first MRI no later than 4 months after birth in patients with achondroplasia. This timing is because the youngest of our patients who showed T2 HSI on MRI was 5 months old. MRI has been recommended at 1 month of age recently [18]. After the initial MRI, it would be good to perform follow-up MRIs at appropriate intervals (e.g., 8 and 12 months of age). Second, we propose the measurement of ‘FM AP’, ‘Cord constriction ratio’, and ‘Posterior indentation’ grade based on the MRIs performed at each follow-up, and the evaluation of any deviation from the cut-off point in these various predictors (for cervical myelopathy: when ‘FM AP’ is < 8 mm or ‘Cord constriction ratio’ > 0.25; for hydrocephalus: when ‘Frontal horn width’ > 50 mm or ‘Posterior indentation’ is observed). When these conditions appear, we can consider examining the symptoms and signs of cervical myelopathy and hydrocephalus more closely, and we can use these parameters to predict the group of patients who are likely to have problems in the future and consider performing preventive surgery before symptoms or signs occur.

### The use of parameters for determining the treatment method of hydrocephalus

Unlike cervical myelopathy, hydrocephalus tends to progress insidiously rather than acutely, and develop reversible neurological deficits [9, 10, 19–21]. Considering the complications of VP shunts [22], it is appropriate to observe the progression sufficiently and consider surgery when clear symptoms develop.

‘Posterior indentation’, ‘Frontal horn width’ and ‘Evans ratio’ were considered predictors of hydrocephalus. Considering these factors and the anatomical location, ‘Posterior indentation’ of the cord at the level of the foramen magnum may be considered a sign of craniocervical CSF flow obstruction. Other studies described that venous hypertension due to jugular foramen narrowing contributed to the development of hydrocephalus, and the role of CSF flow obstruction by foramen magnum stenosis is relatively small [12, 23, 24]. However, all of these publications were case reports or case series, and clear evidence is lacking. Our findings suggest that FMD, instead of VP shunt, may resolve hydrocephalus in symptomatic patients with ventriculomegaly who have evident ‘Posterior indentation’. Cine MRI is sometimes used [25], but clear evidence is lacking, and this method has the disadvantage of requiring an additional MRI.

Eight of our patients presented with symptoms or signs of chronic hydrocephalus. Four of them had T2 HSI and ‘Posterior indentation’ (grades 1, 1, 2, and 2), and 3 of them had only ‘Posterior indentation’ (grades 1, 2, and 2) in the cord on MRI. Therefore, FMD was performed first. The other patient showed severe ventriculomegaly (‘Frontal horn width’: 67.85 mm; ‘Evans ratio’: 0.513) without any abnormalities in the cord on MRI, and VP shunt was performed. As expected, the outcomes of the 7 patients who underwent FMD were good. The signs of hydrocephalus, such as scalp vein engorgement, tense fontanel, and papilledema, resolved in all patients without additional VP shunt. In one patient, ‘Evans ratio’ decreased from 0.37 to 0.34 after 7 months, and the HC decreased from the 95th percentile to the 75th percentile 32 months after surgery. Similar cases were found in other studies [10, 26].

Our study has some limitations. First, due to the large variation in the time of the patients’ first visits, the time when MRI was performed varied from 5 to 49 months. Second, this study was a retrospective analysis performed with patient outcomes to identify predictors. However, consistency was maintained to some extent in that it was the result of management based on prospective criteria. Third, the number of patients with hydrocephalus was small. Therefore, the analysis requires more patients.

## Conclusion

‘FM AP’ and ‘Cord constriction ratio’ may be used to predict cervical myelopathy, and the most significant predictor was ‘Cord constriction ratio’. Measurement of these factors according to the screening program allows closer patient observation and consideration of preventive surgery in patients with predictors that deviate from the cut-off points. ‘Posterior indentation’, ‘Frontal horn width’ and ‘Evans ratio’ may be used in the prediction of hydrocephalus, and FMD should be considered first in patients with severe ‘Posterior indentation’ with satisfactory results to avoid VP shunt.

## Data Availability

The datasets used and/or analyzed during the current study are available from the corresponding author on reasonable request.

## Abbreviations

HSI: High signal intensity
MRI: Magnetic resonance imaging
VP: Ventriculo-peritoneal
CSF: Cerebrospinal fluid
FMD: Foramen magnum decompression
HC: Head circumference
FM AP: Anterior–posterior diameter of the foramen magnum
FM: Foramen magnum
ROC: Receiver operating characteristic
OR: Odds ratio
AUC: Area under the curve
